# Characterization and Quantification of the Major Bioactive Compounds in Mexican Purple Tomatoes

**DOI:** 10.1007/s11130-024-01182-x

**Published:** 2024-05-06

**Authors:** Andrea Torres, Laura J. Pérez-Flores, Ricardo Lobato-Ortíz, Arturo Navarro-Ocana

**Affiliations:** 1 Crop Physiology Laboratory, Department of Agriculture and Animal Production, Division of Biological and Health, Metropolitan Autonomous University, Xochimilco Campus, 04960 Mexico City, Mexico; 2 Department of Health Sciences Division of Biological and Health Sciences, Metropolitan Autonomous University, Iztapalapa Campus, Mexico City, 09310 Mexico; 3 Department of Genetic Resources and Productivity Postgraduate College, Montecillo Campus, Texcoco, Mexico State 56230 Mexico; 4 Department of Food and Biotechnology, Faculty of Chemistry, Nacional Autonomous University of Mexico, Mexico City, 04510 Mexico

**Keywords:** Purple tomato, Acylated anthocyanins, Phenolic acid, Flavonoids, Carotenoids

## Abstract

The present work carries out a quantitative analysis of the major bioactive compounds found in the native Mexican purple tomatoes. Total phenolic content ranged from 7.54 to 57.79 mg TPC/g DM, total flavonoid content ranged from 1.89 to 16.93 mg TFC/g DM, total anthocyanin content ranged from 0.29 to 2.56 mg TAC/g DM, and total carotenoid content ranged from 0.11 to 0.75 mg TCC/ g DM. In addition, 14 phenolic acids were identified, among which caffeoylquinic acid derivatives were the most abundant compounds with chlorogenic acid concentration up to 9.680 mg/g DM, together with flavonoids, such as rutin and quercetin-hexoxide. The qualitative analysis also showed the presence of 9 acylated anthocyanins and 2 carotenoids with significant functional features. As for anthocyanins, their chemical structures disclosed special structural features: glycosylated anthocyanins exhibited *cis-trans* hydroxycinnamic moieties and petunidin-3-(trans-*p*-coumaroyl)-rutinoside-5-glucoside was reported to be the main anthocyanin, whitin the range of concentrations between 0.160 and 1.143 mg/g DM.

**Graphical Abstract:**

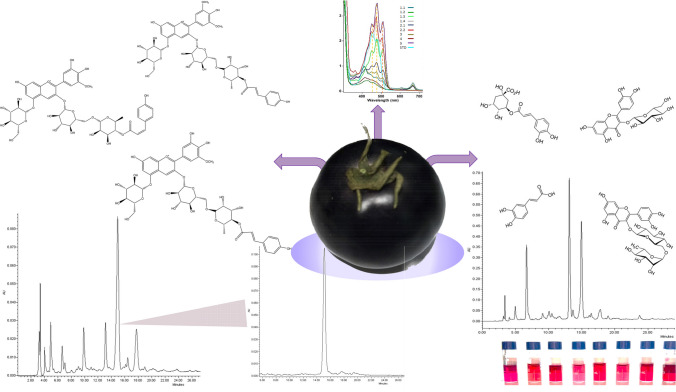

**Supplementary Information:**

The online version contains supplementary material available at 10.1007/s11130-024-01182-x.

## Introduction

Tomato (*Solanum lycopersicum* L*.*) is ranked as the second most important fruit and vegetable crop only preceded by potato (*Solanum tuberosum* L*.*)*.* Tomato is believed to be a native of the areas confined to Peru and Ecuador, but it was domesticated in Mexico, where its cultivated forms were disseminated. Nowadays, tomato is grown all around the world for local consumption or for export [1].

Mainly composed of water (>90%), tomato contains little protein or fat, and approximately 3% (w/w) of carbohydrates (glucose and fructose). As for its morphological structure, pericarp consists of an external layer, namely the exocarp, and mesocarp and endocarp as internal layers. The exocarp (epidermis) consists of a fine cuticle without stomata whose phenolic content increases during fruit growth [2]. The quality of tomato fruits can be determined by assessing the quantity of phytochemicals, sugars, minerals, and vitamins [3]. Common nutrients found in tomatoes include vitamins minerals, fiber, proteins, essential amino acids, monosaturated fatty acids, carotenoids, phytosterols, among others [4]. The presence of carotenoids in tomatoes, such as the content of other bioactive compounds, depends on the fruit genotype and environmental growing conditions. The most abundant carotenoid in tomato is lycopene [5], which confers its characteristic red color, followed by β-carotene which is associated with more orange-yellowish tones. Tomatoes can also vary in color from purple to black, but these tones are directly related to the presence of anthocyanins. In general, these colored phenolics are accumulated in fruit epidermis, and although some authors state that tomatoes do not produce anthocyanins naturally, this characteristic can be obtained by either genetic transformation or by introgression of various species of wild purple fruits [6]. It is also known that the main activity of anthocyanins in fruits is to protect against oxidative stress during fruit development. Moreover, these colored compounds found in tomatoes play a protective role in human body, for example, fighting against oxidative stress, acting as photo protectors and anti-inflammatory agents, to name a few [7]. The anthocyanins detected in purple tomatoes belong to glycosides of delphinidin and petunidin, acylated with coumaric acid, which is typical for most Solanaceae fruits [6]. Color stability is linked to the acyl group that can participate in π stacking interactions with the anthocyanin chromophore (intramolecular co-pigmentation or auto-association), protecting against water molecule addition [8]. However, anthocyanins and other bioactive compounds found in the creole purple tomatoes have been poorly characterized. For that reason, the present study was focused on the identification, characterization and quantification of the major anthocyanins and other bioactive compounds (caffeic acid derivatives, flavonoids, and carotenoids) in different samples of Mexican purple tomato.

## Materials and Methods

### Extraction

Phenolic compounds (TPC, TFC, and TAC) were extracted from 0.5 g of the lyophilized powder of tomato peel (the varieties are described in the SI (supplementary information): plant material and sample preparation and Fig. SI-1), resuspended in 10 mL of the solvent mix that contained methanol: water: lactic acid (80: 19:1, v/v) using ultrasonic bath at 40 °C for 20 min. For the TCC extraction, 0.5 g of the lyophilized powder of tomato peel resuspended in 10 mL of the solvent mix containing hexane: acetone: ethanol (50:25:25, v/v) using ultrasonic bath was sonicated at 25 °C for 20 min. TCC was quantified in the extracts and the results were expressed as mg of bioactive compound/g of dry mass (DM). All the essays were performed in triplicate.

### Quantification of Total Phenol Content (TPC)

The content of phenolic compounds in the samples was determined according to the method previously described by Taga et al. [9]. In brief, 100 μL of the filtrated (described in the extraction section) were mixed with 2 mL of 2% Na_2_CO_3_ (w/v), and the suspension was left to incubate for 2 min. Following that time, 100 μL of Folin-Ciocalteu reagent were added (previously diluted with water at ratio 1:1 (v/v), and this mixture was left to incubate for 30 min. Absorbance was measured at 750 nm using a Cary 60 UV-Vis spectrophotometer. For the calibration curve, standard solutions of gallic acid were prepared in a concentration range from 0.03 to 1.0 mg/mL. Phenols content in extract samples was determined by interpolation on the standard curve, and the results were expressed as mg TPC/ g DM.

### Quantification of Total Flavonoid Content (TFC)

The assay was carried out following the modified method described by Chang [10]. In brief, 1 mL of extract (described in the extraction section) was mixed with 1.4 mL of H_2_O and 300 μL of 5% NaNO_2_ (w/v). The mixture was left to incubate for 5 min. Following that time, 300 μL of 10% AlCl_3_ (w/v), 2 mL of 1 M NaOH, and 5 mL of H_2_O were added. The absorbance was measured at 415 nm. For calibration curve, a series of catechin standard solutions was prepared in a range of concentrations from 0.01 to 0.3 mg/mL. The flavonoid content was determined by interpolation on the calibration curve. The results were expressed as TFC mg/g DM.

### Quantification of Total Anthocyanin Content (TAC)

For that purpose, the differential pH methodology was followed as described by Giusti and Wrolsad [11]. In brief, 1800 μL of 0.025 M potassium chloride buffer pH 1.0 were transferred to a test tube containing 200 μL of extract (described in the extraction section) and stirred. Then, 1800 μL of 0.4 M sodium acetate buffer pH 4.5 were transferred to another test tube containing 200 μL of extract (described in the extraction section) and mixed well. The difference in absorbance between the two samples was measured at 510 and 700 nm. The TAC was calculated using equations proposed by Giusti and Wrolstad:

$$\textrm{TAC}=\left(\textrm{A}\times \textrm{MW}\times \textrm{DF}\times 1000\right)/\left(\upvarepsilon \times 1\right)$$

A: (A_510_ – A_700_) pH 1.0 – (A_510_ – A_700_) pH 4.5
MW: molecular weight
DF: dilution factor
ε: molar absorption coefficient

Molecular weight and molar absorption coefficient used in that formula correspond to those of cyanidin-3-glucoside. The results were expressed as TAC mg/g DM.

### Quantification of Total Carotenoid Content (TCC)

Carotenoid concentrations in test samples was measured according to the method previously described by de Carvalho [12]. In brief, absorbance was measured directly at 450 nm was measured directly in extract solution (described in section of extraction). The following equation was used:

$$\text{TCC}=\left(\text{A}\ast\text{V}\ast10^4\right)/\left(\text{E}\ast\text{p}\;\left(\text{g}\right)\right)$$

A: absorbance at 450 nm
V: Total extract volume
E$${\displaystyle \begin{array}{c}1\%\\ {}1\ cm\end{array}}$$
β-carotene extinction Coefficient in petroleum ether (2592)
p: sample weight

The results were expressed as TCC mg/g DM.

## Results and Discussion

### Quantification of Bioactive Compounds in Purple Tomatoes

The main secondary metabolites (phenols and carotenoids) were analyzed in all nine samples from two regions in Mexico: the Oaxaca State and Metropolitan Zone of the Mexico Valley. The latter samples were sub-grouped into 5 varieties according to the provider source (Fig. SI-1).

**Fig. 1:**
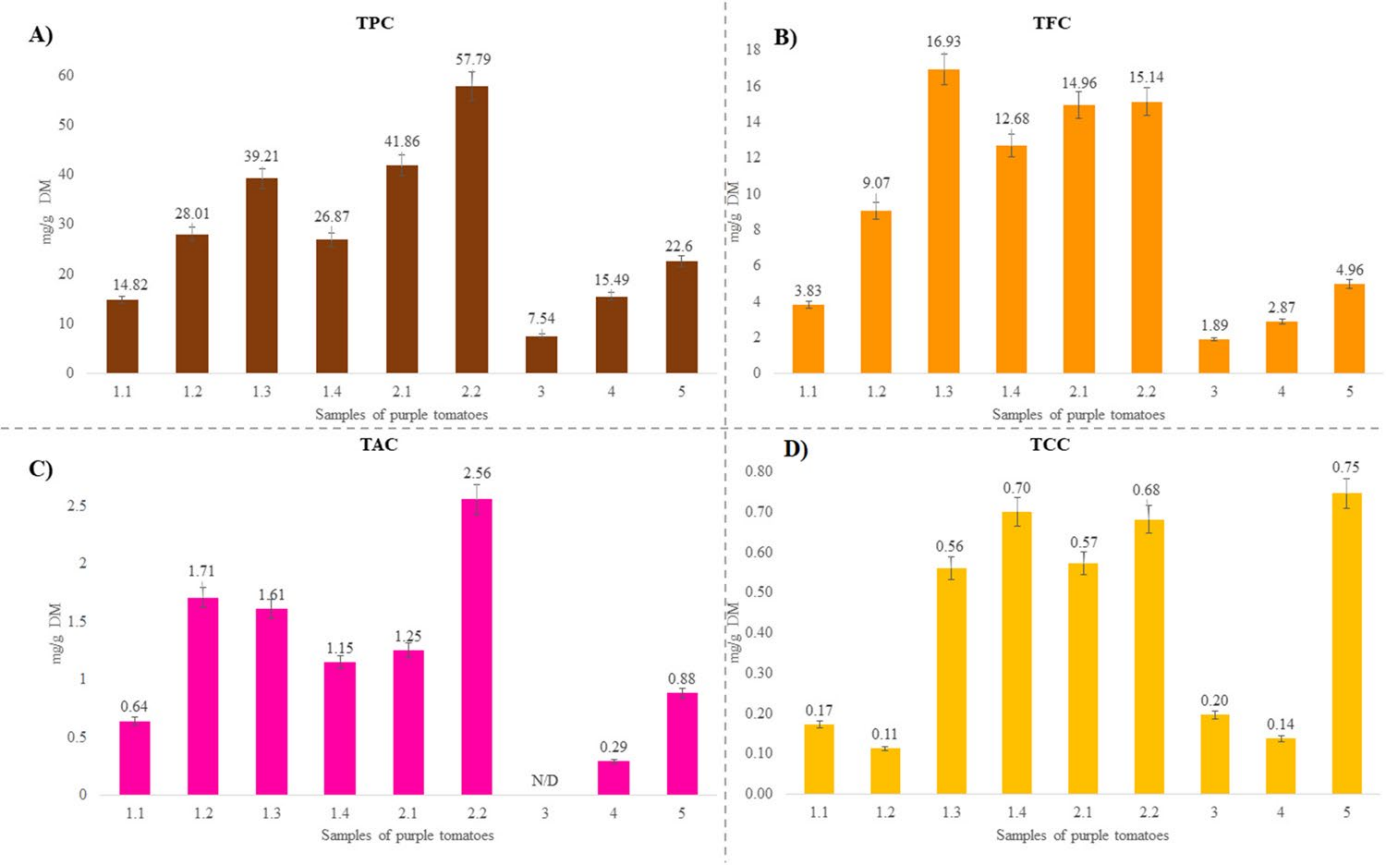
Bioactive compounds in 9 samples of purple tomatoes expressed as mg total bioactive compound/ g purple tomato peel DM. Each bar represents a mean value (*n* = 3)

Since tomato secondary metabolites accumulate mainly in the fruit epidermis, all the analyses performed in this study were limited to the tomato peel. As it can be seen in Fig. 1, the common trait observed for all the test samples regardless their origin was that total phenols (Fig. 1A) comprised the most abundant bioactive compounds identified, with an average content ranging between 7.54–57.79 mg TPC/ g DM. Saladette variety (3) showed the lowest phenol concentrations as well as the other bioactive compounds, followed by the purple indigo (1.1) and the AG Oaxaca (4) and BS Oaxaca (5) varieties. The observed results are comparable with previous studies as it was reported 6.6 mg TPC/g DM for Canadian line V118 of purple tomato [13], and 8.6 mg TPC/g DM for sun Black line in accordance with the fruit ripeness stage. As for flavonoids (Fig. 1B), their concentration was found to be in the range between 1.89–16.93 mg TFC/g DM with a distribution pattern similar to that of TPC. In contrast, anthocyanins (Fig. 1C) were not detected in Saladette variety (3) while their content in other samples ranged from 0.29–2.56 mg TAC/g DM with a similar trend to that observed for TPC. It is worth mentioning that Cherokee varieties (2.1 and 2.2) as well as indigo purple variety (1.2, 1.3 and 1.4) from Mexico City exhibited the highest concentration of anthocyanins, with 2.2 being the highest. A previous study reported anthocyanin contents of 1.2 mg TAC/g DM for genetically modified Sun Black line derived from two wild varieties of purple tomatoes [14]. On the other hand, other authors assessed anthocyanin concentrations in a range from 1.02–1.69 mg/g DM for the Giant purple and New Zealand purple varieties cultivated in Ecuador [15]. Finally, carotenoid content (Fig. 1D) ranged between 0.11 and 0.75 mg TCC/g DM, with the highest total content observed in the BS Oaxaca (5), 2.2 (Cherokee variety) and 1.4 (indigo purple variety). In comparison to previous works, similar values were reported for purple tomatoes (0.45 mg TCC/g DM) and orange cherry tomatoes (0.22 mg TCC/g DM) by Luciano et al. [16]. These authors point out that purple tomatoes synthesize a greater amount of carotenoids than orange tomatoes, attributing this to the genetic mutations that the purple tomato suffered to produce anthocyanins, which apparently promoted carotenoid biosynthesis.

### Identification of Bioactive Compounds in Purple Tomato

**Fig. 2:**
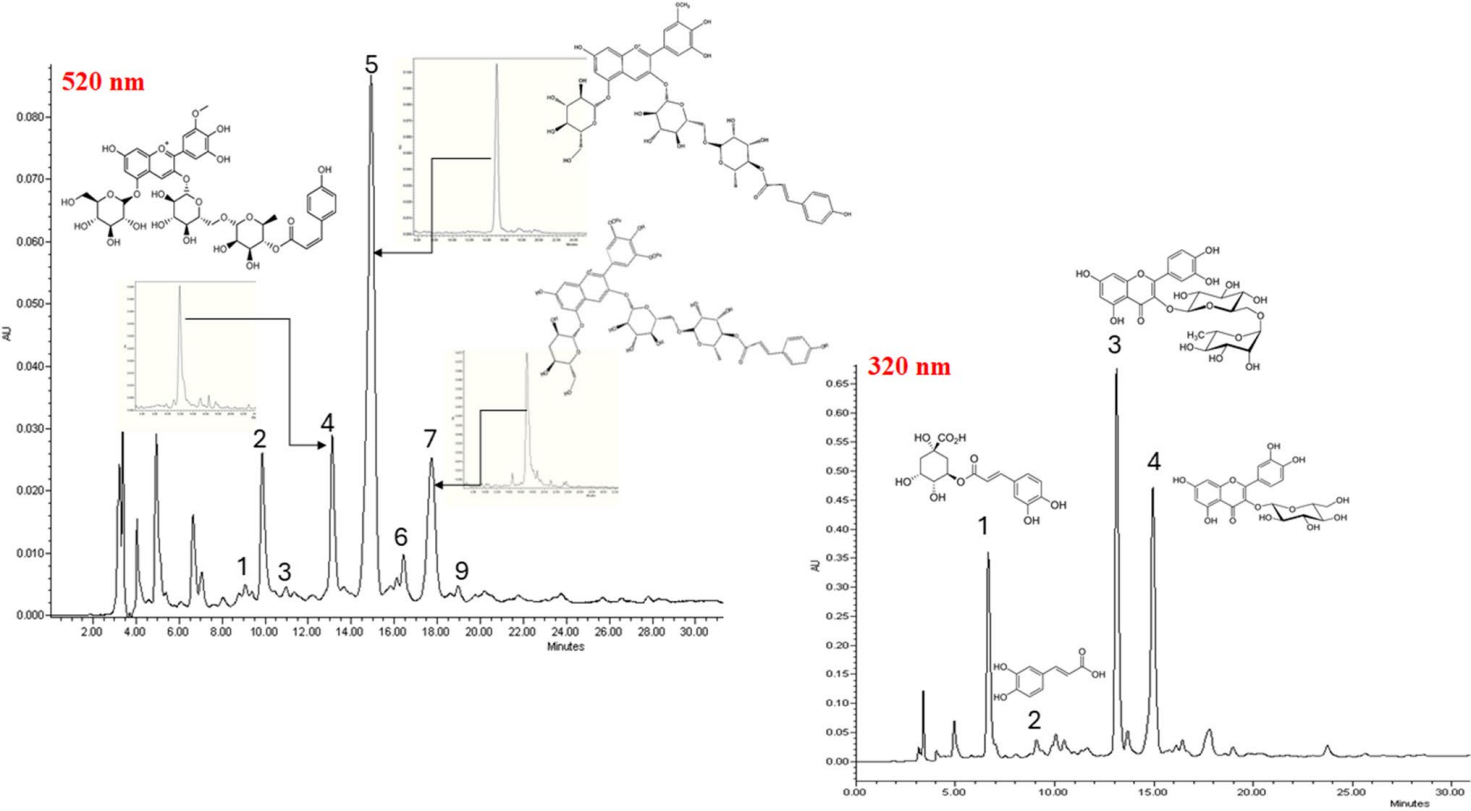
HPLC chromatogram(520 nm) of the of purple tomato extract and chemical structures of the purified anthocyanins and HPLC chromatogram (320 nm) of the of purple tomato extract and chemical structures of the major phenolic compounds

All nine samples were analyzed using HPLC-MS at 320 nm (method described in the SI: HPLC and HPLC-MS analysis). By comparing their retention times and molecular weights, 14 phenolic compounds were identified as shown in Table SI-1 and Fig. 2 (320 nm). The most abundant phenolics were caffeoylquinic acid derivatives, caffeic acid and flavonoids. Among caffeoylquinic acid derivatives, were detected 3 isomers of caffeic acid, 2 isomers of di-caffeoylquinic acid, and feruloyl-caffeoylquinic acid were detected, while flavonoids comprised rutin, quercetin-hexoxide, kaempferol-rutinoside, dihydroxy-dimethoxychalcone-C-diglucoside, and rutin-pentoside. Caffeoylquinic acid derivatives and rutin were present in all nine samples, and the Cherokee variety showed the highest diversity of phenolic acids and flavonoids identified in this study. These phenolic compounds have been previously reported in fresh tomato fruits [17], reported up to 38 phenolic compounds including phenolic acids, hydroxycinnamoyl-quinic acids, derivatives of flavone, flavonol, flavanone and dihydrochalcone.

Regarding colored phenolics, the anthocyanins content was analyzed in 8 samples of purple tomato as shown in Table SI-2 and Fig. 2 (520 nm) (sample 3 was discarded from the analysis since no anthocyanins were detected). HPLC-MS analysis (method described in the SI: HPLC and HPLC-MS analysis) was used to identify 9 anthocyanins with different aglucones (petunidin, delphinidin and malvidin) and acylated with caffeic, ferulic, and coumaric acids. The obtained m/z values matched with the molecular weights of anthocyanin structures previously reported for transgenic purple tomatoes [18] and hybrids [19]. In the present study, these mono-acylated anthocyanins showed a very diverse pattern depending on the variety in test samples. For example, only 4 anthocyanins were identified in the BS Oaxaca (5) variety, while 5 anthocyanins were detected in AG Oaxaca (4). In comparison, up to 6 anthocyanins were quantified in the indigo purple varieties, with Cherokee varieties showing the highest diversity among anthocyanins detected (8 structures). It is worth mentioning that petunidin-3-(trans-*p*-coumaroyl)-rutinoside-5-glucoside, petunidin-3-(cis-*p*-coumaroyl)-rutinoside-5-glucoside, and malvidin-3-(trans-*p*-coumaroyl)-rutinoside-5-glucoside were present in all samples tested.

Finally, the identification of the major carotenoids in the purple tomato samples was carried out spectrophotometrically as shown in Fig. SI-2 (method described in SI: Identification of the main carotenoids using UV-Vis), and was compared with the absorbency maximum of the β-carotenoid standard (450 nm). These absorbance values are in line with previous reports [20]. Another carotenoid identified in the samples of purple tomato was lycopene, which contents were determined accordance to a previous report, with maximum of absorbance at 504 nm as reported for a red tomato variety [21]. However, no lycopene was detected in samples 1.2 and 4 (corresponding to indigo purple and AG Oaxaca varieties, respectively) using this technique. Importantly, those varieties contained the lowest concentrations of total carotenoids (Fig. 1).

### Quantification of the Major Bioactive Compounds in Purple Tomatoes

Major bioactive compounds found in purple tomatoes were quantified and included two phenolic acids, two flavonoids and three anthocyanins (method described in SI: quantification of major anthocyanins, flavonoids and phenolics). As reported in Table 1, the most abundant compound found in all samples was chlorogenic acid in concentrations of up to 0.090–9.680 mg/g DM, followed by rutin 0.250–6.063 mg/g DM. While caffeic acid, and quercetin-hexoside, which were detected at lower concentrations whith a range of up to 0.050 mg/g DM and 1.970 mg/g DM, respectively. The same trend was reported by other authors for different tomato samples with colors varying from red-yellow to purple, where chlorogenic acid was the most abundant phenolic compound [22, 23] followed by rutin [13] (predominant flavonoid in the tomato [24]). Among quantified anthocyanins (4, 5, and 7), petunidin-3-(trans-*p*-coumaroyl)-rutinoside-5-glucoside (Anthocyanin 5) was present at the highest concentration ranging from 0.160 to 1.143 mg/g DM, as seen in the Table 1, which stands for approximately 40% of total anthocyanins detected in each sample (Fig. 1C) and with majority of more stable *trans*-hydroxycinnamic versus *cis*-hydroxycinnamic substituent [25]. Similarly, petunidin-3-(trans-*p*-coumaroyl)-rutinoside-5-glucoside was reported to be the main anthocyanin present in a variety known as Rose indigo with contents up to 2.7 mg/g DM in the fruit epidermis [26]. In contrast, transgenic variety Del/Rose 1, produced up to 3.3 mg/g DM in a fruit peel [18]. It is worth noting that those improved, selected, and transgenic purple tomatoes contain slightly more than twice the amount of total anthocyanins reported in the present study. Other anthocyanins quantified in the present study were present at lower concentrations, varying from sample to sample, for example, malvidin-3-(*trans*-*p*-coumaroyl)-rutinoside-5-glucoside (Anthocyanin 7) was found in a range of 0.016–0.353 mg/g DM while petunidin-3-(*cis*-*p*-coumaroyl)-rutinoside-5-glucoside (Anthocyanin 4) was determined in a concentation range of 0.070–0.213 mg/g DM. The latter structure is considered to be the rarest *cis* enantiomer found in nature [27].

**Table 1:** Phenolic compounds concentration in different samples of purple tomato

Sample	Chlorogenic acid (mg/g)	Caffeic acid (mg/g)	Rutin (mg/g)	Quercetin-hexoside (mg/g)	Ant 5 (mg/g)	Ant 7 (mg/g)	Ant 4 (mg/g)
1	8.307 ± 0.911^B^	0.030 ± 0.003^B^	1.843 ± 0.221^D^	0.073 ± 0.009^E^	0.350 ± 0.052^C^	0.064 ± 0.009^CD^	0.113 ± 0.020^C^
1.2	9.680 ± 1.121^A^	0.024 ± 0.003^C^	3.230 ± 0.434^B^	1.493 ± 0.243^B^	0.733 ± 0.083^B^	0.260 ± 0.041^B^	0.158 ± 0.021^B^
1.3	9.677 ± 1.102^A^	0.050 ± 0.006^A^	6.063 ± 0.652^A^	1.970 ± 0.237^A^	1.097 ± 0.111^A^	0.287 ± 0.033^B^	0.167 ± 0.024^B^
1.4	4.920 ± 0.536^D^	0.020 ± 0.002^C^	2.380 ± 0.219^C^	0.710 ± 0.081^C^	0.437 ± 0.064^C^	0.113 ± 0.024^C^	0.0989 ± 0.001^CD^
2.1	2.237 ± 0.219^F^	0.012 ± 0.002^CD^	3.027 ± 0.441^B^	0.380 ± 0.053^CD^	0.410 ± 0.055^C^	0.100 ± 0.013^C^	0.213 ± 0.029^A^
2.2	5.620 ± 0.623^C^	0.040 ± 0.006^A^	5.980 ± 0.729^A^	2.197 ± 0.341^A^	1.14 ± 0.121^A^	0.353 ± 0.041^A^	0.193 ± 0.023^AB^
3	0.090 ± 0.011^G^	N/D	0.250 ± 0.031^F^	N/D	N/D	N/D	N/D
4	4.830 ± 0.522^D^	0.010 ± 0.002^D^	0.890 ± 0.129^E^	0.193 ± 0.023^DE^	0.170 ± 0.021^D^	0.020 ± 0.003^E^	0.080 ± 0.001^CD^
5	3.417 ± 0.394^E^	0.020 ± 0.003^C^	0.850 ± 0.119^E^	0.210 ± 0.033^DE^	0.160 ± 0.013^D^	0.016 ± 0.002^E^	0.070 ± 0.001^D^

^a^Data are shown as mean value ± SD (standard deviation), *n* = 3, ^b^ Different capital letters in superscript indicate statistically important difference between compounds. N/D (no detected)

### Conclusion

The present study revealed the content of the major bioactive compounds identified in 5 varieties of the purple tomatoes native to Mexico. Those analyzed included phenolic compounds, flavonoids, anthocyanins, and total carotenoids, with 14 phenolic acids and flavonoids, 9 acylated anthocyanins, and 2 carotenoids identified. Anthocyanins with special structural features like glycosylated anthocyanins were also present with *cis-trans* hydrocinnamic substituents. Chlorogenic acid was the most abundant phenolic compound with concentrations up to 9.680 mg/g DM, while petunidin-3-(trans-*p*-coumaroyl)-rutinoside-5-glucoside was the major anthocyanin found at the concentration ranging from 0.160 to 1.143 mg/g DM.

### Supplementary Information

ESM 1 (DOCX 1490 kb)

## Data Availability

No datasets were generated or analyzed during the current study.

## Abbreviations

DM: Dry matter
TAC: Total anthocyanin content
TFC: Total flavonoid content
TPC: Total phenol content
TCC: Total carotenoid content
AG Oaxaca: atomic grape variety
BS Oaxaca: black strawberry variety
HPLC: High*-*performance liquid chromatography
HPLC-MS: High*-*performance liquid chromatography and mass spectrometry
Ant: Athocyanin
