# Supplementary material for: Characterization and Quantification of the Major Bioactive Compounds in Mexican Purple Tomatoes
Source: Plant Foods Hum Nutr. 2024 May 6;79(2):330–6. doi: 10.1007/s11130-024-01182-x (PMC11178568; doi:10.1007/s11130-024-01182-x)
Supplement: Supplementary file 1 — (DOCX 1490 kb) [file 11130_2024_1182_MOESM1_ESM.docx]

Characterization and quantification of the major bioactive compounds in Mexican purple tomatoes

Andrea Torres^a^, Laura J. Pérez-Flores^b^, Ricardo Lobato-Ortíz^c^, Arturo Navarro-Ocana^d*^

_a_ Crop Physiology Laboratory, Department of Agriculture and Animal Production, Division of Biological and Health, Metropolitan Autonomous University- Xochimilco Campus, 04960, Mexico City, Mexico.

_b_ Department of Health Sciences Division of Biological and Health Sciences, Metropolitan Autonomous University- Iztapalapa Campus, 09310, Mexico City, Mexico.

_c_ Department of Genetic Resources and Productivity Postgraduate College – Montecillo Campus, 56230, Texcoco, Mexico State, Mexico.

^d^ Department of Food and Biotechnology, Faculty of Chemistry, Nacional Autonomous University of Mexico, 04510, Mexico City, Mexico.

*Indicates corresponding authors. E-mail: [arturono@unam.mx](mailto:arturono@unam.mx), Tel: 52 55 56225346, https://orcid.org/0000-0002-8538-3631

**SUPPLEMENTARY INFORMATION**

**Material and Methods**

**Plant material and sample preparation**

Samples of purple tomatoes were obtained from the central region (Mexico City and the State of Mexico) and southern Mexico (Oaxaca) and were labelled according to the supplier source: (1) indigo purple variety:1.1 the greenhouses of COLPOS (State of Mexico), 1.2 the greenhouses of Bosque de San Juan de Aragón (Mexico City), 1.3 Xochimilco (Mexico City), 1.4 Oaxaca (San Juan Market, Mexico City); (2) Cherokee purple variety: 2.1 Oaxaca (San Juan Market, Mexico City) and 2.2 the greenhouses of “Los Pinos” cultural complex (Mexico City); (3) saladet coffee variety (The greenhouses of COLPOS (State of Mexico)); (4) atomic grape variety (AG Oaxaca the greenhouses of Oaxaca state) and (5) black strawberry variety (BS Oaxaca the greenhouses of Oaxaca state). The greenhouses provided 10-15 pieces of purple tomatoes from three different plants in 4 harvest cycles and the samples were stored in paper bags at -80 °C in ultra-freezer (Model ELT-13V-85 A 30) prior lyophilization process (Free Zone freeze dryer 4.5). Once dehydrated, exocarp (tomato peel) was separated from endocarp (pulp), ground, and sieved through 60 mesh. The resulting powder was stored in amber glass bottles for further analysis.


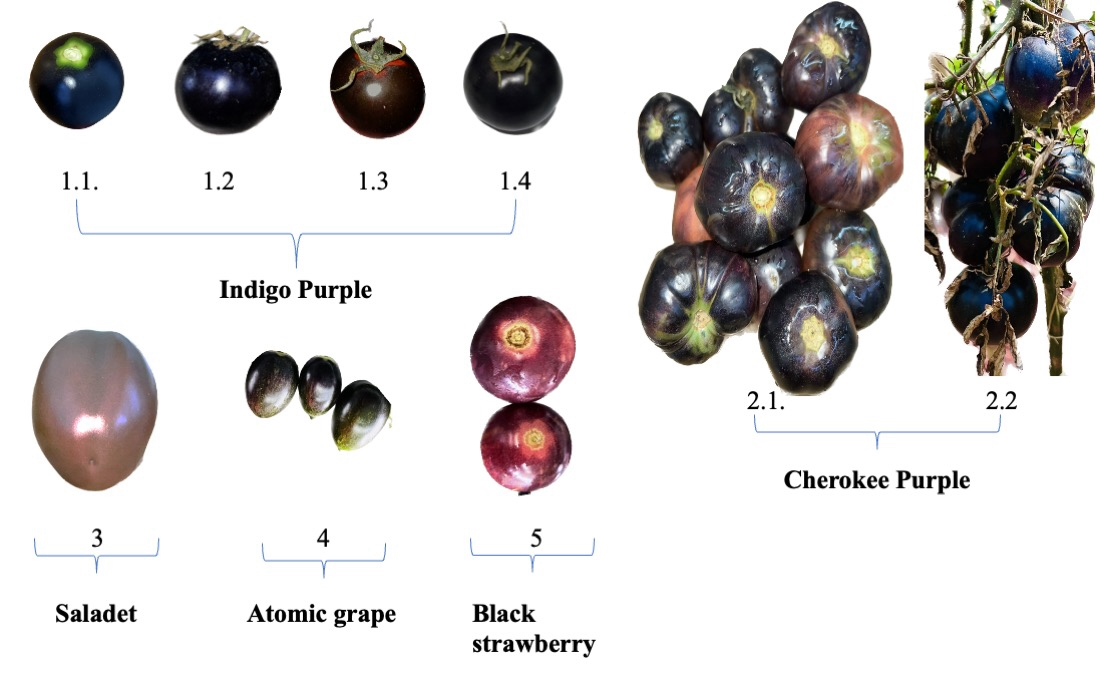


**Fig SI-1** Images of the purple tomato variety samples analyzed in this work.

**Identification of major carotenoids using UV-Vis**

To identify β-carotene and lycopene according to their maximum of absorbance, the extracts used for quantification of total carotenoids were subjected to UV-vis scan in a wavelength range from 200 to 700 nm at 30 °C using a Cary 60 spectrophotometer equipped with multiple cells support and temperature controller.

**Anthocyanins purification**

The phenolic compounds were extracted (described in the extraction section) by ultrasonic bath (Cole-Parmer 8892) at 100 W and 40 °C for 20 minutes using 3 g of the lyophilized tomato peel powder from a purple tomato variety with the highest TAC according to the previous analysis [1]. The respective powdered sample was suspended in 60 mL of the solvent mixture containing methanol: water: lactic acid (48: 19: 1 v/v), and subsequently it was filtered off. The filtrate was concentrated under reduced pressure (Buchi rotary evaporator) and dissolved in 20 mL of H_2_O. Then, 60 mL of hexane were added, and the aqueous fraction was recovered; to which 60 mL of ethyl acetate were added and the aqueous fraction was recovered (fraction enriched with anthocyanins).

Afterwards, amberlite XAD-7HP was added to the aqueous fraction of the purple tomato peel extract (until all the anthocyanins are retained in the resin) and 5 consecutive washes were performed with water. Finally, the adsorbed anthocyanins were eluted from amberlite XAD-7HP with a mixture of methanol: acetic acid (99:1 v/v). The resulting extract, called crude anthocyanin extract, was dried under reduced pressure and the obtained powder was stored at 0 °C [2]. To obtain high purity anthocyanins, a semi-preparative HPLC separation was carried out. A Hypersil Gold C18 selectivity column (250x10 mm; 5 µm) was used and a mobile phase was delivered at a flow rate of 2.5 mL/min. The HPLC elution conditions were as described in Section HPLC analysis. The crude anthocyanin extract powder was dissolved in mobile phase at a concentration of 50 mg/mL and 30 µL were spiked to the column. The purified anthocyanin was dried under reduced pressure (Buchi rotary evaporator) and stored at 0 °C [3].

**HPLC analysis**

The HPLC analysis was performed on a Hypersil Gold C18 selectivity column (250 x 4.6 mm; 5 μm) using Waters system equipped with autosampler and UV-Visible detector (320 nm for phenolic acid and flavonoids, and 510 nm for anthocyanins). The binary mobile phase consisted of (A) water/acetonitrile/formic acid with volume ratio of 89: 10: 1 v/v and (B) acetonitrile that was delivered at a flow rate of 1.0 mL/min and a gradient of 3-25% B in 45 min, 30% B at 47 min, and 3 % B at 56 min [4].

**HPLC-MS analysis**

The identification and characterization of the phenolic compounds and anthocyanins were carried out by means of HPLC-MS as described previously. An Agilent 6410 Triple Quad LC/MS system equipped with a G1311A binary pump, G1316A thermostat column, and G1367E auto-sampler with an electrospray ionization (ESI) source was used. Separation was achieved using a Hypersil-Gold column (250 x 4.6 mm, 5 μm) at 25 ºC. The binary mobile phase consisted of (A) water/acetonitrile/formic acid of 89:10:1 v/v and (B) acetonitrile delivered at a flow rate of 1.0 mL/min and gradient of 97-75 % A in 45 min, 70 % A in 1 min, and 97 % A in 9 min. For MS detection, the negative ion mode was used for phenolic acids and flavonoids while the positive ion mode was applied for anthocyanins. Spectra were registered for m/z between 200 and 2000. Other MS acquisition parameters were as follows: capillary voltage = 4000 V; nebulizer gas (nitrogen) = 50 psi; dry gas flow rate = 12 L/min; source heater dry gas temperature = 350 ºC. The mass spectrometer was programmed to perform a full scan (MS) and a zoom scan of the 933 and 947 m/z ions on the first scan (MS^2^). The ions were monitored with a collision energy of 15 eV (Table 1) [4].

**Quantification of major anthocyanins, flavonoids and phenolics**

To prepare HPLC standard curves, 3 anthocyanins previously purified were weight off with precision and dissolved in 80% methanol (v/v) to obtain 1 mg/mL concentration of mother solution for each test compound. Similarly, mother solutions of 1% chlorogenic acid, caffeic acid, rutin and quercetin-hexoxide (w/v) were also prepared in 80% methanol (v/v) using commercial standards with HPLC grade (Sigma-Aldrich). Equation curves and standard error of regression corresponding to each compound were as follows:

Y _ant5_= 8*10^6^X-47742 R^2^=0.9988

Y _ant7_= 1*10^7^X-6448 R^2^=0.9997

Y _ant4_= 4*10^7^X-88811 R^2^=0.9942

Y _Rut_ = 1*10^7^X-88104 R^2^=0.9998

Y _Quer-hex_ = 2*10^7^X-11598 R^2^=0.9998

Y _Ac clor_= 3*10^7^X+91120 R^2^=0.9993

Y _Ac caf_ = 2*10^8^X+4415 R^2^=0.9981

**Statistical analysis**

All the experiments were performed in triplicate. The results are presented as mean ± standard error of mean. For statistical analysis, one-way ANOVA was used followed by Duncan's multiple comparison post hoc test. Differences were considered statistically significant at P <0.05.

**Results**

**Table SI-1** Identification of phenolic compounds in purple tomato samples according to retention times as determined by HPLC at 320 nm.

| RT  (min) | Compound | PM  (M-1) | Iones  (m/z) | Sample | | | | | | | | |
| --- | --- | --- | --- | --- | --- | --- | --- | --- | --- | --- | --- | --- |
|  |  |  |  | 1.1 | 1.2 | 1.3 | 1.4 | 2.1 | 2.2 | 3 | 4 | 5 |
| 4.9 | 4-cafeoylquinic acid | 353 | 191 | **+** | **+** | **+** | **+** | **+** | **+** | **+** | **+** | **+** |
| 5.7 | Cafeoyl hexoside acid | 341 | 179 | **+** | **+** | **-** | **+** | **+** | **+** | **+** | **-** | **-** |
| 5.9 | 3-cafeoylquinic acid | 353 | 191 | **+** | **+** | **+** | **+** | **+** | **+** | **+** | **+** | **+** |
| **6.8** | **5-cafeoylquinic acid** | **353** | **191** | + | + | + | + | + | + | + | + | + |
| 8.1 | Coumaroyl-hexoside acid | 325 | 187 | **+** | **+** | **+** | **+** | **+** | **+** | **-** | **+** | **-** |
| **9.2** | **Caffeic acid** | **179** | **135** | + | + | **+** | + | + | + | **-** | + | + |
| 10.3 | Rutin-pentoside | 741 | 609 | **-** | **+** | **-** | **+** | **+** | **+** | **-** | **+** | **+** |
| **13.0** | **Rutin** | **609** | **300** | + | + | + | + | + | + | + | + | + |
| 13.5 | Feruloylquinic acid | 367 | 193 | **+** | **+** | **+** | **+** | **+** | **+** | **-** | **+** | **+** |
| 14.9 | **Quercetin-hexoside** | **463** | **300** | + | + | + | + | + | + | - | + | + |
| 16.1 | Kaempferol-3-rutinoside | 593 | 285 | **+** | **+** | **+** | **+** | **+** | **+** | **-** | **-** | **+** |
| 16.5 | Dihydroxy-dimethoxychalcone-C-diglucoside | 623 | 503 | **+** | **+** | **-** | **+** | **+** | **+** | **-** | **+** | **+** |
| 19.0 | 3,5 di-cafeoylquinic acid | 515 | 353,179 | **+** | **+** | **+** | **+** | **+** | **+** | **+** | **+** | **+** |
| 24.5 | 4,5 di-cafeoylquinic acid | 515 | 353 | **+** | **+** | **+** | **+** | **+** | **+** | **+** | **+** | **+** |

**Table SI-2** Identification of anthocyanins in 9 samples of purple tomatoes according to retention times in HPLC-MS analysis at 520 nm.

| No | Anthocyanin | PM (M+1) | Iones (m/z) | Sample | | | | | | | |
| --- | --- | --- | --- | --- | --- | --- | --- | --- | --- | --- | --- |
|  |  |  |  | 1.1 | 1.2 | 1.3 | 1.4 | 2.1 | 2.2 | 4 | 5 |
| 1 | Petunidin-3-(cafeoyl)-rutinoside-5-glucoside | 949 | 787 | + | + | + | + | + | + | - | + |
| 2 | Delphinidin-3-(feruloyl)-rutinoside-5-glucoside | 949 | 787 | - | - | - | - | + | + | - | - |
| 3 | Delphinidin-3**-(**p-coumaroyl)-rutinoside-5-glucoside | 919 | 757 | - | + | + | + | + | + | + | - |
| 4 | **Petunidin-3-(*cis-p-*coumaroyl)-rutinoside-5-glucoside** | **933** | **771** | + | + | + | + | + | + | + | + |
| 5 | **Petunidin-3-(*trans-p*-coumaroyl)-rutinoside-5-glucoside** | **933** | **771, 479, 317** | + | + | + | + | + | + | + | + |
| 6 | Malvidin-3-(*cis-p*-coumaroyl)-rutinoside-5-glucoside | 947 | 785 | - | - | - | - | + | + | - | - |
| **7** | **Malvidin-3-(*trans-p*-coumaroyl)-rutinoside-5-glucoside** | **947** | **785, 493, 331** | + | + | + | + | + | + | + | + |
| 8 | Petunidin 3-(*p*-coumaroyl)-rutinoside | 771 | 479 | + | + | - | - | - | + | - | - |
| 9 | Delphinidin-3-(*p*-cafeoyl)-rutinoside-5-glucoside | 935 | 773 | + | + | + | + | + | + | + | - |

**Fig SI-2** UV-Vis scan of the purple tomato samples: maximum of absorbance for beta-carotene (yellow pointed line using β-carotene standard) and lycopene (orange pointed line)

References:

1. Fernandez‐Aulis F, Hernandez‐Vazquez L, Aguilar‐Osorio G, et al (2019) Extraction and Identification of Anthocyanins in Corn Cob and Corn Husk from Cacahuacintle Maize. J Food Sci 84:954–962. https://doi.org/10.1111/1750-3841.14589

2. Fernandez-Aulis F, Torres A, Sanchez-Mendoza E, et al (2020) New acylated cyanidin glycosides extracted from underutilized potential sources: Enzymatic synthesis, antioxidant activity and thermostability. Food Chem 309: https://doi.org/10.1016/j.foodchem.2019.125796

3. Torres A, Aguilar-Osorio G, Camacho M, et al (2021) Characterization of polyphenol oxidase from purple sweet potato (Ipomoea batatas L. Lam) and its affinity towards acylated anthocyanins and caffeoylquinic acid derivatives. Food Chem 356:. https://doi.org/10.1016/j.foodchem.2021.129709

4. Torres A, Basurto F, Navarro-Ocana A (2019) Quantitative Analysis of the Biologically Active Compounds Present in Leaves of Mexican Sweet Potato Accessions: Phenols, Flavonoids, Anthocyanins, 3,4,5-Tri-Caffeoylquinic Acid and 4-Feruloyl-5-Caffeoylquinic Acid. Plant Foods for Human Nutrition 74:. https://doi.org/10.1007/s11130-019-00774-2
